# Metabolic Bariatric Surgery in the Era of GLP-1 Receptor Agonists for Obesity Management

**DOI:** 10.1001/jamanetworkopen.2024.41380

**Published:** 2024-10-25

**Authors:** Kevin Lin, Ateev Mehrotra, Thomas C. Tsai

**Affiliations:** 1 Department of Health Care Policy, Harvard Medical School, Boston, Massachusetts; 2 Department of Health Services, Policy and Practice, Brown School of Public Health, Providence, Rhode Island; 3 Department of Surgery, Brigham and Women’s Hospital, Boston, Massachusetts

## Abstract

This cross-sectional study investigates rates of bariatric surgery and glucagon-like peptide-1 receptor agonist prescription among adults without diabetes in 2022 to 2023.

## Introduction

Use of glucagon-like peptide-1 receptor agonists (GLP-1 RAs) semaglutide and liraglutide as antiobesity medications has surged in recent years.^1,2^ Anecdotally, health systems have closed hospital-based metabolic bariatric surgery programs due to decreased demand, but empirical data on the association of increased prescribing of GLP-1 RAs with use of metabolic bariatric surgery is unavailable, to our knowledge. We assessed national trends and characteristics of patients with obesity who were prescribed GLP-1 RAs compared with those undergoing metabolic bariatric surgery.

## Methods

In this cross-sectional study, we used 2022 to 2023 claims from 16.8 million unique deidentified adult patients with medical and pharmaceutical coverage through commercial and Medicare Advantage insurance in the OptumLabs Data Warehouse. We included only patients without diabetes. Similarly, we included only GLP-1 RA prescriptions with FDA indications as antiobesity medications (eMethods in Supplement 1). For each quarter, we identified all patients with any claim for any formulation of semaglutide or liraglutide and for metabolic bariatric surgery (eMethods in Supplement 1).

We compared enrollee-level characteristics, including age, sex, and Elixhauser comorbidity index, among patients prescribed GLP-1 RAs and who underwent metabolic bariatric surgery during the study period.^3^ We used χ^2^ tests for statistical comparison.

We then assessed trends in use of GLP-1 RAs and metabolic bariatric surgeries per 1000 unique patients without diabetes using a generalized linear regression model. Due to seasonal variations in metabolic bariatric surgery use, we compared the slope of change during quarter 3 and 4 between 2022 and 2023.^4^ This study was approved by the institutional review board at Harvard Medical School and deemed exempt from informed consent due to minimal risk. Results are reported in accordance with the STROBE reporting guideline, and 2-sided P values <.05 were considered statistically significant. Analyses were performed in SAS statistical software version 9.4 (SAS Institute).

## Results

During the study period, 83 346 patients (0.5%) were prescribed GLP-1 RAs and 9863 patients (0.01%) underwent metabolic bariatric surgery. Patient characteristics for the year 2023 are shown in the Table. Patients with metabolic bariatric surgery were more medically complex than those prescribed GLP-1 RAs or no treatment (17.9% vs 6.9% vs 4.7% with ≥4 comorbidities; *P* < .001) (Table).

**Table.** Characteristics of Patients Without Diabetes Prescribed GLP-1 RA, Undergoing Metabolic Bariatric Surgery, or Neither in 2023

Characteristic	Patients, No. (%) (N = 13 448 870)^a^			*P* value
	Metabolic bariatric surgery (n = 4662)	GLP-1 RA prescription (n = 58 193)	Neither treatment (n = 13 386 015)^b^	
Age, y				
18-35	863 (17.9)	6976 (12.0)	3 718 580 (27.8)	<.001
36-50	1624 (33.6)	16 683 (28.6)	2 928 520 (21.9)	
51-65	1151 (23.8)	17 323 (29.7)	2 637 300 (19.7)	
≥66	1024 (21.2)	17 211 (29.5)	4 101 620 (30.6)	
Sex^c^				
Female	3681 (76.2)	44 858 (76.9)	6 939 920 (51.8)	<.001
Male	977 (20.2)	13 294 (22.8)	6 435 320 (48.1)	
Unknown	4 (0.1)	41 (0.1)	10 780 (0.1)	
Comorbidities, No.				
0	482 (10.0)	17 397 (29.8)	7 867 380 (58.8)	<.001
1	1171 (24.2)	19 333 (33.1)	2 932 120 (21.9)	
2-3	2146 (44.4)	17 420 (29.8)	1 952 940 (14.6)	
≥4	863 (17.9)	4043 (6.9)	633 580 (4.7)	

Abbreviation: GLP-1 RA, glucagon-like peptide-1 receptor agonist.

^a^ Adult patients without diabetes and medical and pharmaceutical coverage in 2023 were included. A total of 169 individuals were excluded who received both a GLP-1 RA and metabolic bariatric surgery in 2023 to prevent duplication across groups. (Numbers in the table reflect values after these 169 individuals were excluded.)

^b^ Subgroups within the neither treatment category do not sum to the provided n value due to rounding. The true number of enrollees who received neither treatment is given, but for space and processing efficiency considerations, subgroup analysis was conducted on a 5% sample, then multiplied by 20, estimating numbers for each subgroup. Similarly, the unknown sex N is estimated to be 900, when the true number of enrollees with unknown sex was 45.

^c^ Across all 3 groups, 11 573 individuals had an unknown sex (ie, were not labeled male or female).

We identified a relative 105.6% increase in patients prescribed GLP-1 RAs between the last 6 months of 2022 vs the last 6 months of 2023 (2.16 vs 4.44 patients per 1000 patients). In contrast, there was a relative 8.7% decrease in patients undergoing metabolic bariatric surgery comparing the same periods (0.23 vs 0.21 patients per 1000 patients). The Figure shows rates for each individual quarter.

**Figure.**
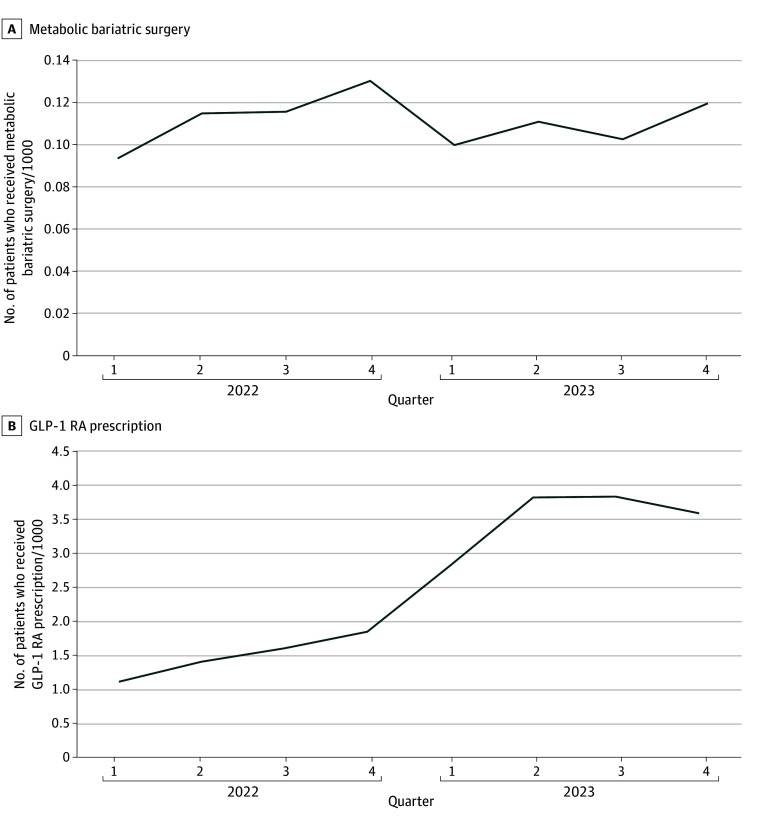
Quarterly Trends in Obesity Treatment, 2022-2023 Glucagon-like peptide-1 receptor agonist (GLP-1 RA) and metabolic bariatric surgery use are tracked across 2022 to 2023 quarters. The number of patients who received a metabolic bariatric surgery per 1000 individuals (A) and the number of patients prescribed a GLP-1 RA per 1000 individuals (B) are shown.

## Discussion

This cross-sectional study of privately insured patients found a more than 2-fold increase in use of GLP-1 RAs as antiobesity medications from 2022 to 2023, with a relative 8.7% decrease in the rate of metabolic bariatric surgery during the same period. Our results provide a national contemporaneous estimate of the decline in metabolic bariatric surgery associated with the era of GLP-1 RAs.

Although GLP-1 RAs are effective for the treatment of obesity and related comorbid conditions, such as diabetes, the high cost and high rates of gastrointestinal adverse effects can lead to treatment cessation and subsequent weight regain.^5^ Further data are needed to assess whether trends in metabolic bariatric surgery use will stabilize with ongoing access barriers to GLP-1 RAs. Our findings also suggest a remaining large addressable market for obesity treatment, with 0.5% of patients without diabetes receiving GLP-1 RAs and 0.01% receiving surgery. Limitations of this study include its cross-sectional nature, changing population denominator secondary to insurance status, and potential confounding from variations in patient adherence to GLP-1 RAs. Policymakers and clinicians should continue to closely monitor trade-offs between pharmacologic and surgical management of obesity to ensure optimal access to effective obesity treatment.

## Notes

Supplement 1.
eMethods.

Supplement 2.
Data Sharing Statement

## References

[1] Brown C. High price and demand for semaglutide means lack of access for US patients. BMJ. 2023;382:1863. doi:10.1136/bmj.p1863 37648269

[2] Watanabe JH, Kwon J, Nan B, Reikes A. Trends in glucagon-like peptide 1 receptor agonist use, 2014 to 2022. J Am Pharm Assoc (2003). 2024;64(1):133-138. doi:10.1016/j.japh.2023.10.002 37821008

[3] Quan H, Sundararajan V, Halfon P, . Coding algorithms for defining comorbidities in *ICD-9-CM* and *ICD-10* administrative data. Med Care. 2005;43(11):1130-1139. doi:10.1097/01.mlr.0000182534.19832.83 16224307

[4] Chhabra KR, Fan Z, Chao GF, Dimick JB, Telem DA. The role of commercial health insurance characteristics in bariatric surgery utilization. Ann Surg. 2021;273(6):1150-1156. doi:10.1097/SLA.0000000000003569 31714318

[5] Sodhi M, Rezaeianzadeh R, Kezouh A, Etminan M. Risk of gastrointestinal adverse events associated with glucagon-like peptide-1 receptor agonists for weight loss. JAMA. 2023;330(18):1795-1797. doi:10.1001/jama.2023.19574 37796527 PMC10557026

