# Coding and Duplication Errors

**DOI:** 10.1001/jamanetworkopen.2025.1603

**Published:** 2025-02-19

Coding and duplication errors occurred in the study that was published as the Research Letter “Metabolic Bariatric Surgery in the Era of GLP-1 Receptor Agonists for Obesity Management”^1^ in *JAMA Network Open* on October 25, 2024. The authors explain the errors and corrections needed in a Comment.^2^ The corrections affect the text, Table, Figure, and Supplement 1. The main findings were reduced in magnitude but did not change direction, and the interpretations and conclusions are not affected. The article has been corrected.^1^
