# Supplementary material for: Metabolic Bariatric Surgery in the Era of GLP-1 Receptor Agonists for Obesity Management
Source: JAMA Netw Open. 2024 Oct 25;7(10):e2441380. doi: 10.1001/jamanetworkopen.2024.41380 (PMC11581531; doi:10.1001/jamanetworkopen.2024.41380)
Supplement: Supplement 1. — eMethods. [file jamanetwopen-e2441380-s001.pdf]

# Supplemental Online Content

Lin K, Mehrotra A, Tsai TC. Metabolic bariatric surgery in the era of GLP-1 receptor agonists for obesity management. *JAMA Netw Open*. 2024;7(10):e2441380.
doi:10.1001/jamanetworkopen.2024.41380

**eMethods.**

This supplemental material has been provided by the authors to give readers additional information about their work.

© 2024 Lin K et al. *JAMA Network Open.*

**eMethods**

*Identifying of Metabolic Bariatric Surgery Cases*

We used the following CPT Codes to identify metabolic bariatric surgery cases: 43633, 43644, 43645, 43659, 43770, 43775, 43842, 43843, 43844, 43845, 43846, and 43847.

*Identifying Patients With Diabetes*

We used ICD10 codes from E10 through E11 to identify patients with diabetes.

*Classifying Comorbidities*

Elixhauser Comorbidity Index was calculated within the OptumLabs Data Warehouse that identifies comorbidities based off diagnosis codes. For this analysis, we looked for the following comorbidities: congestive heart failure, cardiac arrhythmias, valvular disease, pulmonary circulation disorders, peripheral vascular disorders, uncomplicated hypertension, complicated hypertension, hypothyroidism, liver disease, metastatic cancer, and depression. We excluded the following comorbidities: paralysis, other neurological disorders, chronic pulmonary disease, uncomplicated diabetes, complicated diabetes, renal failure, peptic ulcer disease excluding bleeding, AIDS/HIV, lymphoma, solid tumor without metastasis, rheumatoid arthritis, coagulopathy, obesity, weight loss, fluid and electrolyte disorders, blood loss anemia, deficiency anemia, alcohol abuse, drug abuse, and psychoses.

*GLP-1 Receptor Agonists Included*

We searched for pharmacy claims for GLP-1 semaglutide and liraglutide, generic names. Below is a table of GLP-1 prescriptions for nondiabetic patients across brands in 2023. We included all

© 2024 Lin K et al. *JAMA Network Open.*

formulations of semaglutide and liraglutide in order to account for potential off-label prescribing of drugs like Ozempic, which has recently had broad media coverage as a "weight loss drug."[6]

| Brand Name | Generic Name |
|---|---|
| Ozempic | semaglutide |
| Rybelsus | semaglutide |
| Saxenda | liraglutide |
| Victoza 2-PAK | liraglutide |
| Victoza 3-PAK | liraglutide |
| Wegovy | semaglutide |
| Xultophy 100-3.6 | liraglutide |

© 2024 Lin K et al. *JAMA Network Open.*
