# Supplementary material for: Metabolic Bariatric Surgery in the Era of GLP-1 Receptor Agonists for Obesity Management
Source: JAMA Netw Open. 2024 Oct 25;7(10):e2441380. doi: 10.1001/jamanetworkopen.2024.41380 (PMC11581531; doi:10.1001/jamanetworkopen.2024.41380)
Supplement: Supplement 2. — Data Sharing Statement [file jamanetwopen-e2441380-s002.pdf]

Data Sharing Statement

Lin. Metabolic Bariatric Surgery in the Era of GLP-1 Receptor Agonists for Obesity Management. *JAMA Netw Open*. Published October 25, 2024. doi:10.1001/jamanetworkopen.2024.41380

Data
Data available: No
